# Effective approach to organic acid production from agricultural kimchi cabbage waste and its potential application

**DOI:** 10.1371/journal.pone.0207801

**Published:** 2018-11-20

**Authors:** Ho Myeong Kim, Ji Hye Park, In Seong Choi, Seung Gon Wi, Sanghyun Ha, Ho Hyun Chun, In Min Hwang, Ji Yoon Chang, Hak-Jong Choi, Jin-Cheol Kim, Hae Woong Park

**Affiliations:** 1 R&D Division, World Institute of Kimchi, Gwangju, Republic of Korea; 2 Asian Pear Research Institute, Chonnam National University, Gwangju, Republic of Korea; 3 Division of Applied Bioscience & Biotechnology, Chonnam National University, Gwangju, Republic of Korea; Agricultural University of Athens, GREECE

## Abstract

The biotransformation of agricultural waste into valuable chemicals represents a promising approach in the field of biorefining. Herein, a general but highly efficient and robust process is reported for the production of organic acid from kimchi cabbage waste using lactic acid bacteria. The organic acid produced was tested for efficacy as a biological control agent. *Lactobacillus sakei* WiKim31 and *L*. *curvatus* WiKim38 could efficiently produce organic acids including lactic acid (12.1 and 12.7 g/L), fumaric acid (7.4 and 7.1 g/L), and acetic acid (4.5 and 4.6 g/L) from kimchi cabbage waste (3% substrate loading, w/v) by simultaneous saccharification and fermentation processes for 48 h, and the culture filtrate induced complete mortality of J2s *Meloidogyne incognita* at 2.5% concentration. These results suggested that lactic acid bacteria *L*. *sakei* WiKim31 and *L*. *curvatus* WiKim38 can efficiently produce organic acids, and the culture filtrate can be applied as a microbial nematicide.

## Introduction

Agricultural waste is recognized as refuse and defined as the unwanted materials or byproducts generated from various agricultural activities, which account for over 30% of worldwide agricultural productivity [1]. However, agricultural waste contains a large amount of carbohydrates and various bioactive compounds and can therefore be harnessed as a useful resource in the biorefining industry (chemicals, energy, foods, medicines, etc.) [2,3]. The recycling of waste materials using the bioconversion process has become a major component of environmental protection, which involves reducing the amount of carbon dioxide produced. As a result, sustainable or green chemistry has been a primary focus to improve the quality of life and health for humans.

According to a Food and Agricultural Organization (FAO) report, the annual global production of cabbage and other brassicas increased to 105.7 million tons in 2014 because of their useful medicinal effects and consumption as food. Specifically, kimchi (traditional Korean food) derived from kimchi cabbage (*Brassica rapa* subsp. *pekinensis*), known as *baechu* in Korea, has anti-cancer, anti-diabetes, and anti-obesity effects [4]. However, in South Korea, a considerable amount of waste is generated because of the overproduction of kimchi cabbage and byproducts from the kimchi manufacturing process. Numerous studies have focused on the production of various bio-based products from agricultural waste [5–7]. In particular, cruciferous vegetable residue contains high fermentable sugar content, including sucrose, glucose, and fructose [8]. Therefore, kimchi cabbage waste (KCW) may become a useful resource in biomaterial production and for agricultural applications, which require high amounts of sugar, organic matter, and vitamins; thus, it may be exploited in the production of organic acids, bio-sugar, biofuel, and organic fertilizer [9].

In general, lactic acid bacteria (LAB) were isolated and identified from the various sources (milk, fermented food, plant, and fruit etc.). LAB as gram positive bacteria mainly divided two groups, *i*.*e*., homofermentrs and heterofermenters, based on the major end-products [10]. Homofermenters mainly produce lactic acid whereas heterofermenters produce several kinds of products (organic acid, carbon dioxide, and ethanol, etc). Therefore, the selection of appropriate LAB strains is required for the production of organic acid.

Organic acids are widely used in the chemical, food, cosmetic, pharmaceutical, and beverage industries owing to their various functional properties [11]. Furthermore, the nematicidal effect of organic acids in the field of biological control has been extensively reported [12,13]. The global organic acid market was estimated to reach $6.94 million by 2016 and is expected to increase annually to $12.54 billion by 2026, based on the organic acid market analysis report [14]. In general, organic acids are produced by chemical synthesis, which results in serious environmental pollution worldwide. Therefore, process optimization and development of high-efficiency bacterial strains are indispensable for organic acid production using eco-friendly biotransformation of agricultural waste.

Organic acid production from biomass resources has been well established in previous studies [15,16]. Herein, organic acid production from KCW using LAB and comparison of separate hydrolysis and fermentation (SHF) and simultaneous saccharification and fermentation (SSF) processes are reported. Finally, potential applications of the produced organic acid in the field of biological control are suggested.

## Materials and methods

### Optimization of carbon and nitrogen sources for LAB growth

LAB (*Weissella cibaria* WiKim28, *Lactobacillus sakei* WiKim31, and *L*. *curvatus* WiKim38) were obtained from the World Institute of Kimchi in Gwangju, South Korea. To select the optimal carbon source, a submerged culture of LAB was conducted in a 10-mL volume containing 2% carbon source (glucose, fructose, galactose, sucrose, maltose, lactose, sorbitol, xylitol, mannitol, soluble starch, corn starch, potato starch, or sweet potato starch), 2.0% yeast extract, 0.5% sodium acetate, 0.2% dipotassium phosphate, 0.01% magnesium sulfate, and 0.005% manganese sulfate for 24 h at 30°C. For determination of growth ability, culture samples were diluted and then poured on MRS plates. After 48 h of incubation at 30°C, the number of colonies on the plate was counted. The optimal nitrogen source (yeast extract, beef extract, soy bean meal, soy powder, ammonium nitrate, or sodium nitrate) was selected under the same conditions and volumes as described above.

### Raw materials and chemical composition analysis

KCW was obtained from a kimchi cabbage field in Haenam, South Korea. The KCW was dried by a lyophilizer at -80°C for 5 days and then ground to particles by an electric grinder. The sample was stored at -20°C until further use. The water-soluble sugar content of the KCW was analyzed using high-performance liquid chromatography (HPLC) with a refractive index detector (2414; Waters, Milford, MA, USA) and REZEX RPM (Phenomenex, Torrance, CA, USA) column (300 mm × 7.8 mm), which was used at 85°C by adding deionized water at a flow rate of 0.6 mL min^–1^. The water-insoluble sugar content of the KCW was analyzed using gas chromatography. The pretreatment and analysis method was performed as described by Choi et al. [17].

### Histochemical analysis for pectin

For the analysis of the general anatomy of kimchi cabbage, sections of embedded tissue of kimchi cabbage were stained with 0.05% Toluidine blue O (prepared in 1% borax), rinsed with water, and observed with light microscopy. Histochemical analysis of fresh and recently collected kimchi cabbage was performed on transverse sections that were cut with razor blades. Two different staining procedures were used to visualize pectins. Specifically, ruthenium red and hydroxylamine–ferric chloride staining were used for identification of unesterified and methyl esterified pectins, respectively [18,19]. In brief, sections were stained with 0.02% aqueous ruthenium red solution for 5 min and mounted with a drop of 50% glycerol. For hydroxylamine–ferric chloride staining, sections were soaked in a freshly prepared mixture of 14% hydroxylamine hydrochloride (in 60% ethanol) and 14% sodium hydroxide (in 60% ethanol) for 10 min. An equal volume of concentrated HCl was added for 1 to 2 min and then replaced with a 10% ferric chloride solution (0.1 N HCl). Light microscopy was carried out on a Zeiss Axiolab microscope (Carl Zeiss, Hallbergmoss, Germany).

### Optimization of enzyme loading content

Cellulase (Celluclast 1.5 L) and pectinase (Pectinex SP-L) were purchased from Novozyme A/S (Bagsvaerd, Denmark) for enzymatic hydrolysis of KCW. The cellulose activity was measured with the National Renewable Energy Laboratory (NREL) method [20], and the pectinase activity was determined as described by Kittur et al. [21]. The cellulase and pectinase activities were 0.356 filter paper unit/mg protein and 240 international unit (IU)/mg protein, respectively. To optimize the enzyme loading content, enzymatic hydrolysis was performed on 1% substrate (KCW, w/v) with variable loading of cellulase (0.0 mg/g to 22.4 mg/g of KCW) and pectinase (2.1 mg/g to 16.8 mg/g of KCW) for 24 h at 45°C. After the enzymatic hydrolysis, the reducing sugar content was measured using 3,5-dinitrosalicylic acid reagent and a glucose standard curve [22].

### Enzymatic hydrolysis according to the KCW concentration

To produce the reducing sugar, enzymatic hydrolysis of KCW dry matter (3–15%, w/v) was conducted in 100-mL total volume with citrate buffer (0.05 M, pH 4.8). Based on previously determined optimal enzyme loading content, cellulase of 5.6 mg/g KCW and pectinase of 4.2 mg/g KCW were added to solution and incubated with at 45°C for 48 h in a 500-mL Erlenmeyer flask. After the reaction, reducing sugar content and conversion yield were calculated according to the glucose standard curve and initial sugar content of the KCW, respectively.

### Separate hydrolysis and fermentation (SHF)

The enzymatic hydrolysis of KCW was conducted in a 100-mL total volume containing 1.5–6.0% (w/v) dry matter, cellulase (5.6 mg/g KCW), pectinase (4.2 mg/g KCW), 2.0% yeast extract, 0.5% sodium acetate, 0.2% dipotassium phosphate, 0.01% magnesium sulfate, and 0.005% manganese sulfate at E°C for 24 h. The fermentation of 40 mL hydrolysates (1.5–6.0%) with 0.4 mL LAB—*W*. *cibaria* WiKim28, *L*. *sakei* WiKim31, and *L*. *curvatus* WiKim38, respectively—was performed at 30°C for 6 days. Culture broths of LAB were centrifuged at 8000 rpm (5810R, fixed angle type; Eppendorf, USA), serially diluted, and filtered through a 0.45-μm PTFE syringe filter (Whatman, USA). The content of organic acid was measured by HPLC (Waters Alliance e2695 system, USA) at 30°C using an Aminex HPX-87H column (300 mm × 7.8 mm, Bio-Rad, Hercules, CA, USA). Elution was carried out isocratically using 5 mmol/L sulfuric acid. The flow rate and detection wavelength were 0.6 mL/min and 210 nm, respectively. Quantitative analysis of organic acid was performed using standard curves.

### Simultaneous saccharification and fermentation (SSF)

The SSF processes were conducted for the KCW in a 40-mL total volume containing 1.5–6.0% (w/v) dry matter, cellulase (5.6 mg/g KCW), pectinase (4.2 mg/g KCW), 0.4 mL LAB—*W*. *cibaria* WiKim28, *L*. *sakei* WiKim31, and *L*. *curvatus* WiKim38, respectively—2.0% yeast extract, 0.5% sodium acetate, 0.2% dipotassium phosphate, 0.01% magnesium sulfate, and 0.005% manganese sulfate at 32°C for 6 days. After the reaction, the organic acid content was measured by HPLC.

### Nematode preparation

Tomato roots infected with *Meloidogyne incognita* were obtained from a tomato greenhouse at Chonnam National University in Gwangju, South Korea. The egg masses were picked off the roots and then blended with 0.5% sodium hypochlorite. The eggs in turn were passed through a 63-μm sieve and 25-μm sieve and then washed with distilled water for in vitro experiments. The eggs were incubated at 28°C for 2.5 days and allowed to hatch using a modified Baermann funnel [23].

### Nematicidal effect of culture filtrate on *Meloidogyne incognita* J2s

To evaluate the nematicidal effect of *W*. *cibaria* WiKim28 on *M*. *incognita* J2s, *W*. *cibaria* WiKim28 culture filtrate was prepared at four concentrations of 0.63%, 1.25%, 2.5%, and 5.0% and added to each well of 96-well tissue culture plates (Becton Dickinson, Franklin Lakes, NJ, USA). The mortality test was conducted with approximately 50 J2s at each concentration. Sterile distilled water was used as a negative control. The plates were shaken at 25°C with 100% humidity in the dark. After a 72-h incubation, the live J2s were counted. The mortality rate was calculated using Abbott’s formula [24]:

Mortality(%)=[(mortalitypercentageintreatment−mortalitypercentageinuntreatedcontrol)/(100−mortalitypercentageinuntreatedcontrol)]×100

Similar experiments were performed with *L*. *sakei* WiKim31 and *L*. *curvatus* WiKim38.

### Statistical analysis

Data were analyzed using the PASW software (Ver. 17; SPSS Inc., USA). Analysis of variance (ANOVA) tests were used to determine the significant differences between treatments at p < 0.05 using Tukey’s HSD test.

## Results and discussion

**Fig 1:**
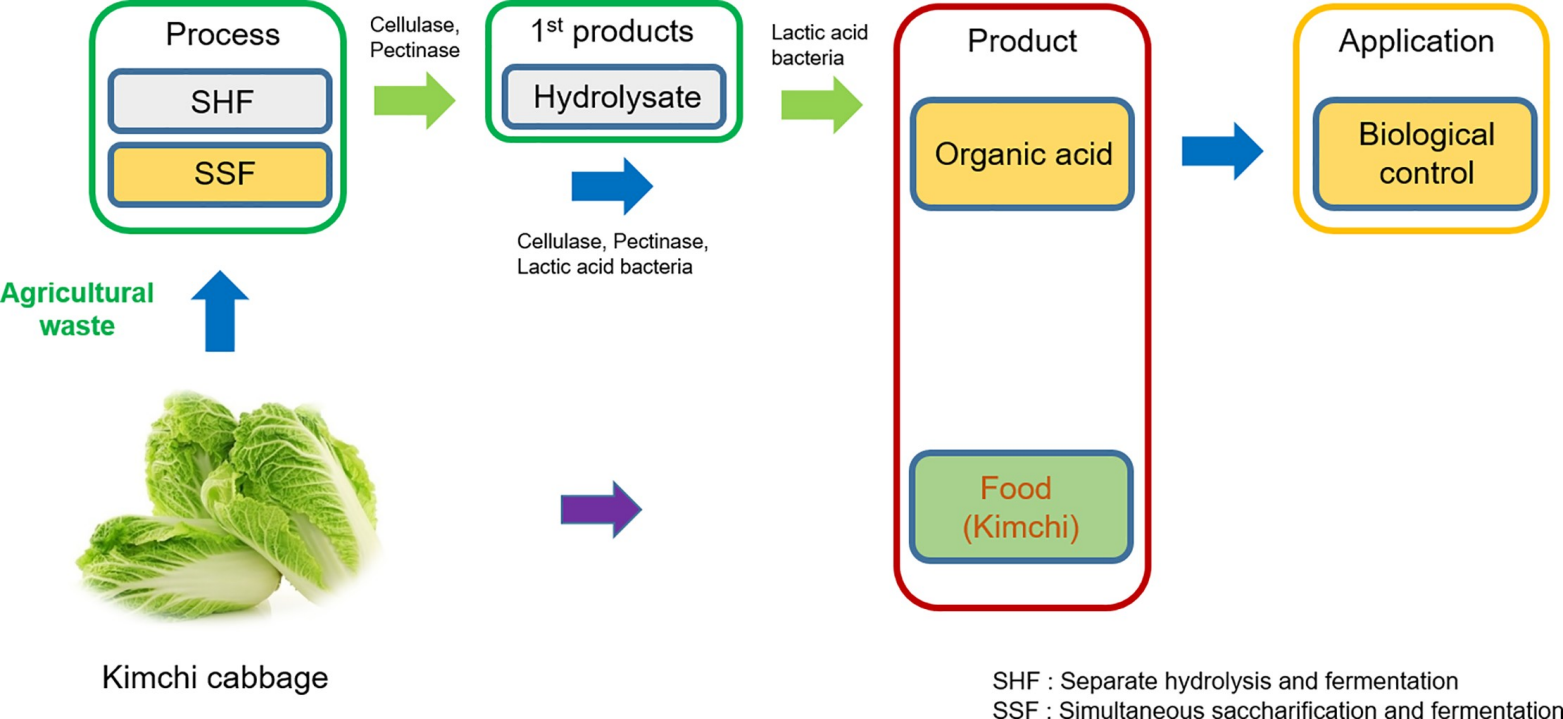
Schematic representation of the utilization of kimchi cabbage waste as a resource.

Kimchi cabbage is one of the major agricultural products cultivated worldwide. In this study, organic acid was produced from KCW, and successful utilization of the organic acid was demonstrated (Fig 1).

### Optimization of carbon and nitrogen source for the growth of LAB

Optimization of the carbon and nitrogen source is necessary to increase cell growth and reduce cost. *W*. *cibaria* WiKim28 and *L*. *curvatus* WiKim38 showed the highest cell mass production—1.9 × 10^9^ CFU/mL and 1.6 × 10^9^ CFU/mL, respectively—when 2% glucose and 2% yeast extract were used as the carbon and nitrogen source, whereas *L*. *sakei* WiKim31 showed the highest cell mass production—2.1 × 10^9^ CFU/mL—when 2% sucrose and 2% yeast extract were used (S1 Fig).

Industrial carbon and nitrogen sources were primarily used for LAB production due to the low price, costing mostly below 3$/kg-carbon source and 10$/kg-nitrogen source, respectively. Media optimization can improve the production yields of LAB cells, resulting in cutting the production cost. Mass production in a conventional industrial fermenter further reduces production cost of LAB.

### Chemical composition analysis of KCW

The carbohydrate content of a biomass resource is highly important in terms of biorefining [25]. Therefore, sufficient carbohydrate content is required to produce fermentable sugars for bio-based application. Agricultural waste has a sufficient carbohydrate content of more than 50%. The chemical composition of KCW is presented in Table 1. This chemical composition was different from the results obtained by Song et al. [8], likely because the carbohydrate content and nutritional value are influenced by the agronomic method, cultivar, maturity stages, harvest time, storage time, and environmental conditions [5, 26]. The KCW had the following main chemical components, in order of concentration: glucose (33.3%), fructose (19.5%), sucrose (1.0%), galactose (1.0%), arabinose (1.0%), xylose (1.0%), and mannose (0.6%). Glucose and fructose were confirmed as the major carbohydrates in the KCW. This result suggested that the sufficient amount of carbohydrates, including glucose and fructose, in KCW allows its use for biotransformation by LAB (*W*. *cibaria* WiKim28, *L*. *sakei* WiKim31, and *L*. *curvatus* WiKim38) for bio-based application.

**Table 1:** Chemical composition of kimchi cabbage waste.

(% dry matter)	Soluble sugar				Insoluble sugar						Total	
	Suc	Glu	Fru	Total	Ara	Xyl	Man	Gal	Glu	Total	Glu	Fru
Kimchi cabbage waste	1.0±0.1	23.4±0.4	19.5±0.3	43.9±0.3	1.0±0.0	1.0±0.1	0.6±0.0	1.0±0.1	9.9±0.4	13.5±0.6	33.3±0.4	19.5±0.3

Values represent the average of three replicates. Suc; sucrose, Glu; glucose, Fru; fructose, Ara; arabinose, Xyl; xylose, Man; mannose, Gal; galactose.

### Histochemical analysis of pectin

The structure and pectin distribution of kimchi cabbage for the selection of hydrolase were analyzed by light microscopy. The pectin heteropolysaccharide is a major component of the middle lamella and primary cell wall of terrestrial plants [27].

**Fig 2:**
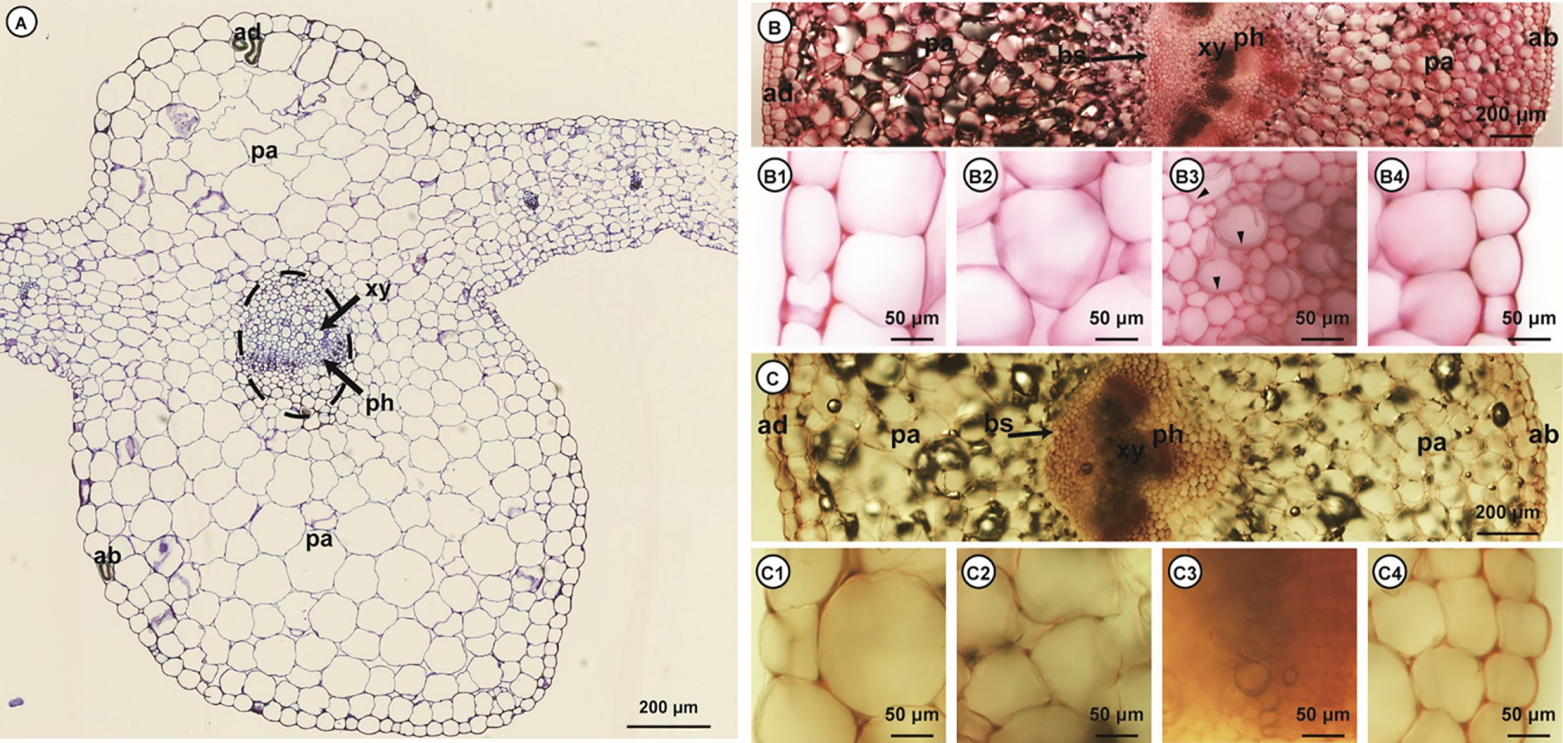
**Histochemical analysis of embedded (A) and hand-cut (B and C) sections of kimchi cabbage.** General view (A). Ruthenium red (B) strongly stained primary walls; the thick secondary walls did not react (arrows). Hydroxylamine–ferric chloride (C) staining in the primary walls but not in the secondary walls. ab, abaxial epidermis; ad, adaxial epidermis; bs, bundle sheath; pa, parenchyma; ph, phloem; xy, xylem.

Fig 2A shows the overall structure of kimchi cabbage. Ruthenium red (which stains unesterified pectin) stained the middle lamella and primary cell walls of the epidermis, parenchyma, xylem, and phloem tissues (Fig 2B). In contrast, the secondary cell walls of the xylem in the vascular bundle were not stained. Similar results were obtained with hydroxylamine–ferric chloride (methyl esterified pectin) staining (Fig 2C). A previous study on onion pectin showed a similar pectin distribution [5]. These results indicate that pectin presented mainly in the primary cell wall of all tissues and not in the secondary cell wall of vessels in the xylem, and kimchi cabbage can be efficiently degraded to fermentable sugar by pectinase.

### Optimization of enzyme loading content

In previous studies, production of biomaterials such as bio-sugar, bioethanol, and organic acid from biomass resources required pretreatment technology such as chemical, physical, physico-chemical, and biological pretreatment [28]. Pretreatment is an important factor for lignocellulosic biomass conversion and is essential to remove the lignin and change the structure of lignocellulosic biomass to make cellulose and hemicellulose fiber more accessible to the enzymes, such as cellulase and xylanase, that convert the cellulose and hemicellulose into monomeric sugars [29]. However, the pretreatment processes increase the cost of the production of biomaterials. Unlike lignocellulosic biomass, KCW is structurally soft with a very low lignin content, which is advantageous because fermentable sugar can be easily produced by enzymatic hydrolysis without a pretreatment process.

**Fig 3:**
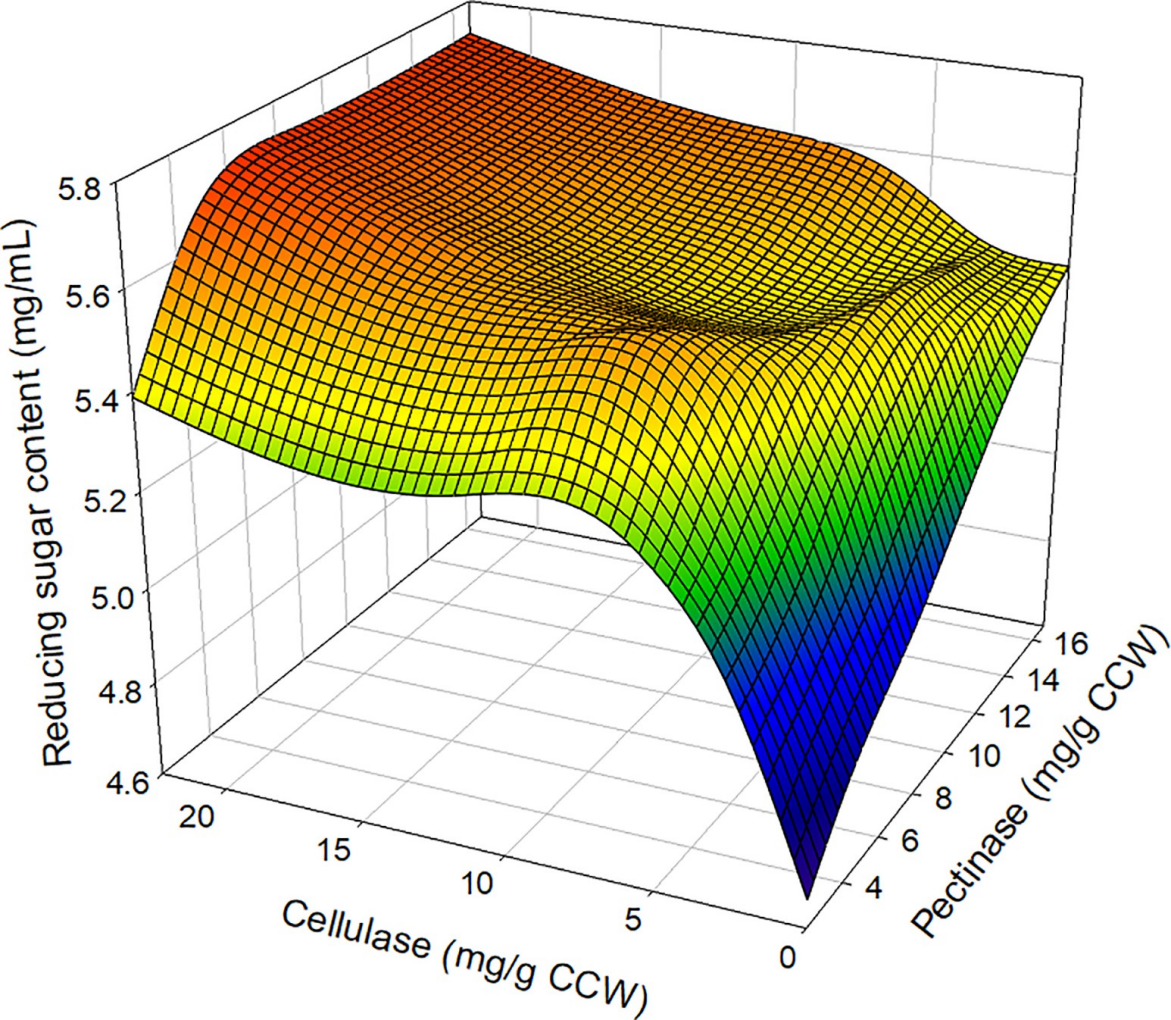
Three-dimensional (3D) profiles of reducing sugar production according to the enzyme loading content.

The optimization of enzyme loading content and enzymatic hydrolysis is a key step in the bioconversion of biomass resources [30]. In this study, KCW was efficiently hydrolyzed to monomeric sugar in the presence of pectinase because commercial pectinase contains various enzymes such as poly-galacturonase, pectin lyase, pectin methylesterase, cellulolytic enzymes, and proteolytic enzymes [31]. Furthermore, the reducing sugar content was increased by the addition of cellulase. After a 24-h reaction, the reducing sugar content was confirmed, and then the optimal loading content for the enzymes was established: 4.2 mg/g KCW of pectinase and 5.6 mg/g KCW of cellulase (Fig 3 and S1 Table).

### Reducing sugar production according to the KCW concentration

KCW is an attractive resource for bio-based application because it has a high carbohydrate content and is easily degraded into monomeric sugar without any pretreatment. For efficient sugar production, enzymatic hydrolysis of KCW (3–15%) was conducted at 45°C for 48 h. In general, substrate loading at high concentration reduces the conversion yield because of substrate inhibition [32,33]. However, the optimization of substrate loading content is required to reduce the cost and obtain high fermentable sugar content. After 48 h of enzymatic hydrolysis, KCW samples presented reducing sugar content of 17.0, 33.9, 48.0, 63.2, and 75.0 g/L and conversion yields of 98.7, 98.4, 93.0, 91.7, and 87.1% for the 3, 6, 9, 12, and 15% substrates, respectively (Table 2). As a result, the conversion yields decreased slightly when substrate concentration was increased from 3% to 15%. In one similar study, the high substrate loading of fruit waste decreased the fermentable sugar production and conversion rate [34].

**Table 2:** Reducing sugar production and conversion yield according to the concentration of KCW.

KCW concentration (%)	24 h		48 h	
	Reducing sugar (g/L)	Conversion yield (%)	Reducing sugar (g/L)	Conversion yield (%)
3.0	16.7±0.36	97.0±2.1	17.0±0.44	98.7±2.5
6.0	33.2±0.72	96.4±2.1	33.9±0.79	98.4±2.3
9.0	46.5±1.44	90.0±2.8	48.0±0.62	92.9±1.2
12.0	59.2±1.47	85.9±2.1	63.2±0.98	91.8±1.4
15.0	67.0±0.92	77.8±1.1	75.0±0.82	87.1±1.0

Values represent the average of three replicates.

### Separate hydrolysis and fermentation (SHF)

To attain economic feasibility of SHF of KCW, high organic acid production efficiency must be achieved. The SHF process can result in a higher production yield compared with that of the SSF process because of the optimization of the enzymatic hydrolysis and fermentation process [35]. Therefore, the enzymatic hydrolysis and fermentation process was conducted at 45°C and 30°C, respectively.

**Fig 4:**
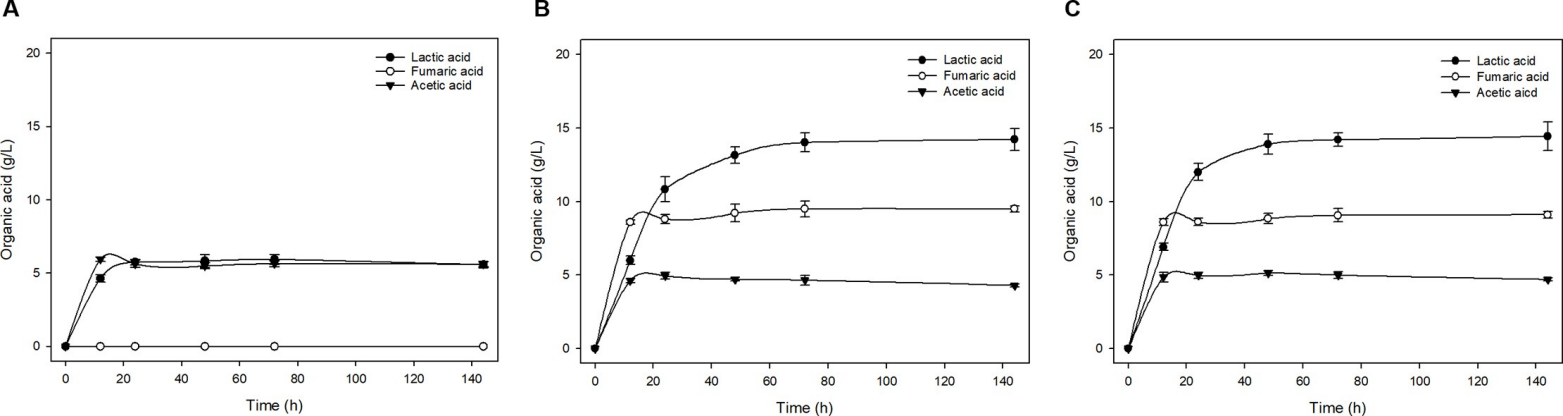
Separate hydrolysis and fermentation (SHF) processes. Time courses of organic acid production for the kimchi cabbage waste (KCW) of 3.0% (dry matter, w/v). (A) *W*. *cibaria* WiKim28. (B) *L*. *sakei* WiKim31. (C) *L*. *curvatus* WiKim38.

The organic acid content was obtained using SHF processes (Fig 4 and S2 Fig**)**. *L*. *sakei* WiKim31 and *L*. *curvatus* WiKim38 mainly produced lactic acid, fumaric acid, and acetic acid, whereas *W*. *cibaria* WiKim28 produced lactic acid and acetic acid. The different substrate concentrations and strains led to differences in the production of organic acid. The organic acids were rapidly produced during the initial 24 h of fermentation, after which production was slowed or maintained (Fig 4). In addition, the organic acid content was effectively increased with KCW hydrolysate of up to 3% (w/v), but the organic acid content decreased or did not significantly differ for the KCW hydrolysate of 4.5% (w/v) and 6.0% (w/v) owing to end-product inhibition [36]. In a previous study, organic acid is known as a negative inhibitor that strongly inhibits cell growth and product formation in the fermentation process [37]. After a 48-h reaction with the KCW hydrolysate of 3.0% (w/v), *L*. *sakei* WiKim31 and *L*. *curvatus* WiKim38 produced lactic acid (13.2 and 13.9 g/L), fumaric acid (9.2 and 8.9 g/L), and acetic acid (4.7 and 5.1 g/L), respectively, whereas *W*. *cibaria* WiKim28 produced lactic acid (8.4 g/L) and acetic acid (4.5 g/L) (Fig 4). As a result, *L*. *sakei* WiKim31 and *L*. *curvatus* WiKim38 efficiently produced organic acid from the KCW hydrolysate of 3.0% (w/v) compared with *W*. *cibaria* WiKim28. These results indicated that *L sakei* WiKim31 and *L*. *curvatus* WiKim38 can be used for the production of organic acid from KCW.

### Simultaneous saccharification and fermentation (SSF)

SSF is recognized as the most common method in the biotechnology field. In fact, SSF can reduce the capital cost, substrate inhibition, and overall process time compared with SHF [38]. In this study, the growth of LAB stains *W*. *cibaria* WiKim28, *L*. *sakei* WiKim31, and *L*. *curvatus* WiKim38 was reduced at temperatures above 35°C, so the SSF processes were conducted at 32°C.

**Fig 5:**
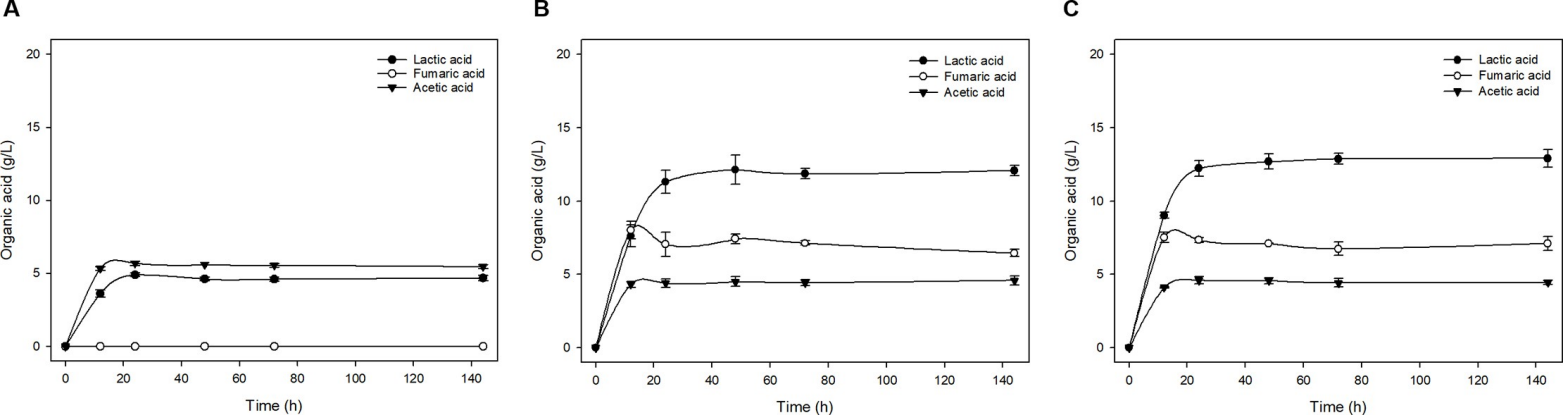
Simultaneous saccharification and fermentation (SSF) processes. Time courses of organic acid production for the kimchi cabbage waste (KCW) of 3.0% (dry matter, w/v). (A) *W*. *cibaria* WiKim28. (B) *L*. *sakei* WiKim31. (C) *L*. *curvatus* WiKim38.

The organic acid content was obtained using SSF processes (Fig 5 and S3 Fig). After 48 h of SSF of 3.0% (w/v) KCW, *L*. *sakei* WiKim31 and *L*. *curvatus* WiKim38 produced lactic acid (12.1 and 12.7 g/L), fumaric acid (7.4 and 7.1 g/L), and acetic acid (4.5 and 4.6 g/L), respectively, whereas *W*. *cibaria* WiKim28 produced lactic acid (4.6 g/L) and acetic acid (5.6 g/L) (Fig 5). The SHF processes showed slightly more efficient production of organic acid than the SSF processes because of the optimization of the enzymatic hydrolysis and fermentation process (*F* = 16.2, *df* = 1,12, *P* < 0.05). However, SSF processes of *L*. *sakei* WiKim31 and *L*. *curvatus* WiKim38 showed a tendency to rapidly produce high amounts of lactic acid with KCW of 4.5% (w/v) and 6.0% (w/v) within 24 h of reaction compared with SHF processes (S2 and S3 Figs). The SSF processes have been extensively studied for the production of organic acid because of the high production yield [39,40]. Consequently, considering the economic feasibility, SSF is considered to be more suitable than SHF for the production of organic acid from KCW.

### Nematicidal effect of culture filtrate

Nematicides are chemical pesticides that are highly toxic to humans and animals [41]. The development of safe and eco-friendly nematicides has become an urgent issue in the field of biological control. Recently, the nematicidal activity of organic acids has been extensively reported [12,13].

**Fig 6:**
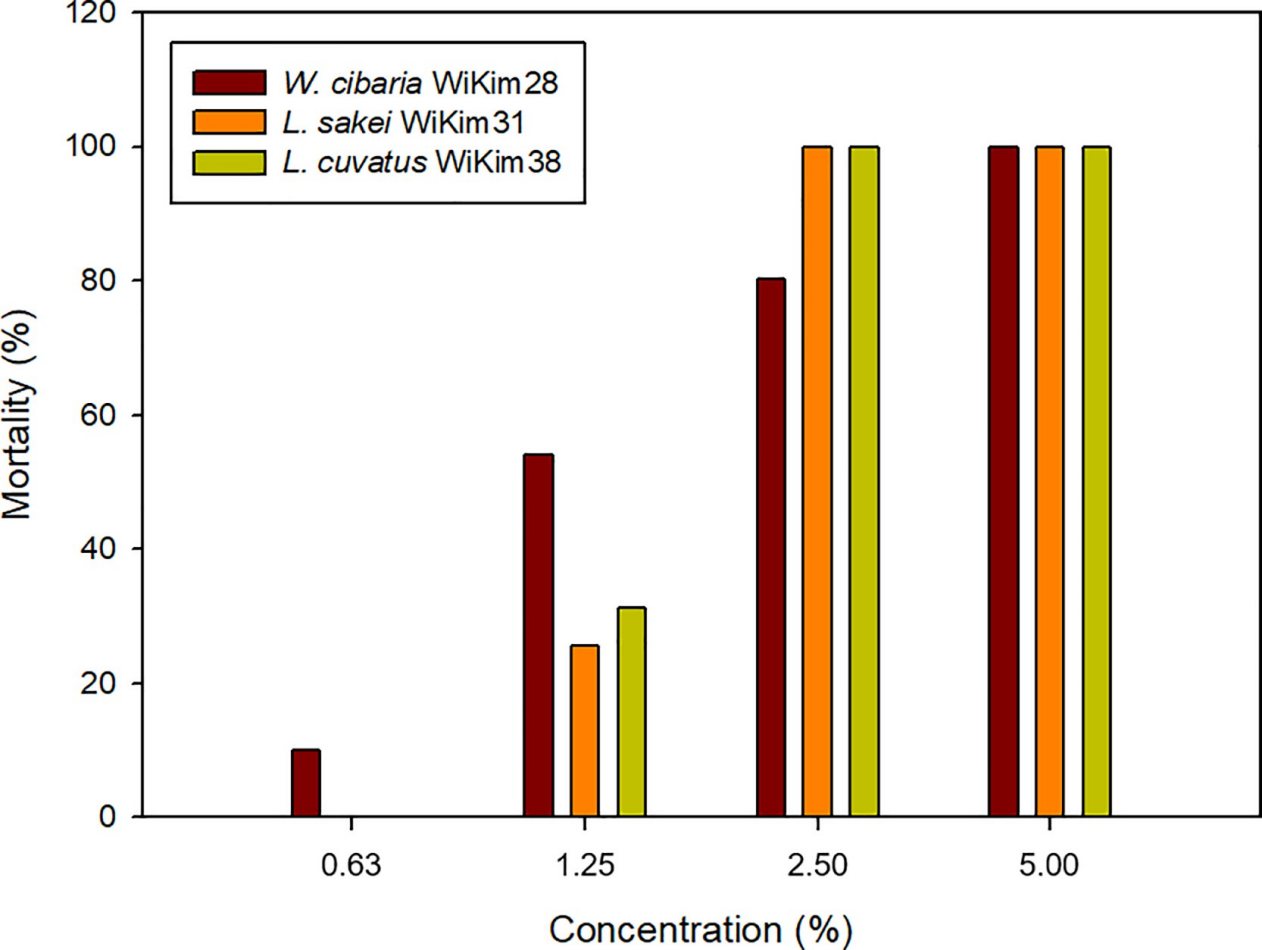
Nematicidal effect of lactic acid bacteria culture filtrate on J2s *M*. *incognita*. The values of mortality represent the averages of three replicates.

Herein, culture filtrates were produced by SSF processes from KCW of 3.0% (w/v) for 48 h. The complete mortality of J2s *M*. *incognita* was confirmed with the 2.5% and 5.0% culture filtrates of *L*. *sakei* WiKim31 and *L*. *curvatus* WiKim38 and with the 5.0% culture filtrate of *W*. *cibaria* WiKim28 (Fig 6). However, the 1.25% culture filtrate of *W*. *cibaria* WiKim28 induced higher J2s mortality (54.1%) compared with those of *L*. *sakei* WiKim31 (25.6%) and *L*. *curvatus* WiKim38 (31.2%). In a similar study, acetic acid showed higher nematicidal activity on *M*. *incognita* J2s compared with lactic acid [42]. In the current study, the 2.5% culture filtrates of *L*. *sakei* WiKim31 and *L*. *curvatus* WiKim38 showed better nematicidal activity compared with that of *W*. *cibaria* WiKim28, regardless of the low concentration of acetic acid, but the produced organic acids are not considered to have a synergistic effect. The culture filtrates of *L*. *sakei* WiKim31 and *L*. *curvatus* WiKim38 also had a higher acid concentration than that of the culture filtrate of *W*. *cibaria* WiKim28 (Table 3). Consequently, this result indicated that the various organic acids in a culture filtrate can increase the nematicidal activity against *M*. *incognita* J2s.

**Table 3:** Final pH value after SHF and SSF processes for 144 h.

Strain	KCW concentration (%)	pH	
		SHF	SSF
*W*. *cibaria* WiKim28	1.5	4.86±0.08	4.85±0.07
	3.0	4.52±0.04	4.50±0.08
	4.5	4.31±0.08	4.46±0.04
	6.0	4.43±0.00	4.45±0.04
*L*. *sakei* WiKim31	1.5	4.64±0.15	4.49±0.00
	3.0	4.06±0.06	4.10±0.08
	4.5	3.98±0.04	4.03±0.11
	6.0	4.01±0.02	3.96±0.06
*L*. *cuvatus* WiKim38	1.5	4.55±0.13	4.46±0.08
	3.0	3.97±0.07	4.06±0.08
	4.5	3.92±0.04	4.00±0.03
	6.0	3.99±0.05	4.06±0.05

Values represent the average of three replicates.

## Conclusions

In this study, the production of organic acid from KCW was evaluated, and the application of the produced organic acid as a biological control agent was demonstrated. The SHF processes were slightly superior to the SSF processes for organic acid production from KCW. In particular, the culture filtrates (2.5% concentration) of *L*. *sakei* WiKim31 and *L*. *curvatus* WiKim38 from SSF processes induced complete mortality of J2s *M*. *incognita*. It was therefore concluded that KCW is a major resource for the production of organic acid, which might be applied in the agricultural sector as a microbial nematicide.

## Supporting information

S1 Fig Production of lactic acid bacteria (LAB) depending on carbon and nitrogen sources. (A) *W*. *cibaria* WiKim28. (B) *L*. *sakei* WiKim31. (C) *L*. *curvatus* WiKim38. (D) *W*. *cibaria* WiKim28. (E) *L*. *sakei* WiKim31. (F) *L*. *curvatus* WiKim38.
(TIF)

S2 Fig Separate hydrolysis and fermentation (SHF) processes. Time courses of organic acid production for the kimchi cabbage waste (KCW) of 1.5, 4.5, and 6.0% (dry matter, w/v). *W*. *cibaria* WiKim28 (A) KCW of 1.5%, (B) KCW of 3.0%, (C) KCW of 6.0%. *L*. *sakei* WiKim31 (D) KCW of 1.5%, (E) KCW of 3.0%, (F) KCW of 6.0%. *L*. *curvatus* WiKim38 (G) KCW of 1.5%, (H) KCW of 3.0%, (I) KCW of 6.0%.
(TIF)

S3 Fig Simultaneous saccharification and fermentation (SSF) processes. Time courses of organic acid production for the kimchi cabbage waste (KCW) of 1.5, 4.5, and 6.0% (dry matter, w/v). *W*. *cibaria* WiKim28 (A) KCW of 1.5%, (B) KCW of 3.0%, (C) KCW of 6.0%. *L*. *sakei* WiKim31 (D) KCW of 1.5%, (E) KCW of 3.0%, (F) KCW of 6.0%. *L*. *curvatus* WiKim38 (G) KCW of 1.5%, (H) KCW of 3.0%, (I) KCW of 6.0%.
(TIF)

S1 Table Reducing sugar concentration of KCW hydrolysate under different enzyme loading content. (DOC)
