# Supplementary figures and images for: Effective approach to organic acid production from agricultural kimchi cabbage waste and its potential application
Source: PLoS One. 2018 Nov 20;13(11):e0207801. doi: 10.1371/journal.pone.0207801 (PMC6245790; doi:10.1371/journal.pone.0207801)

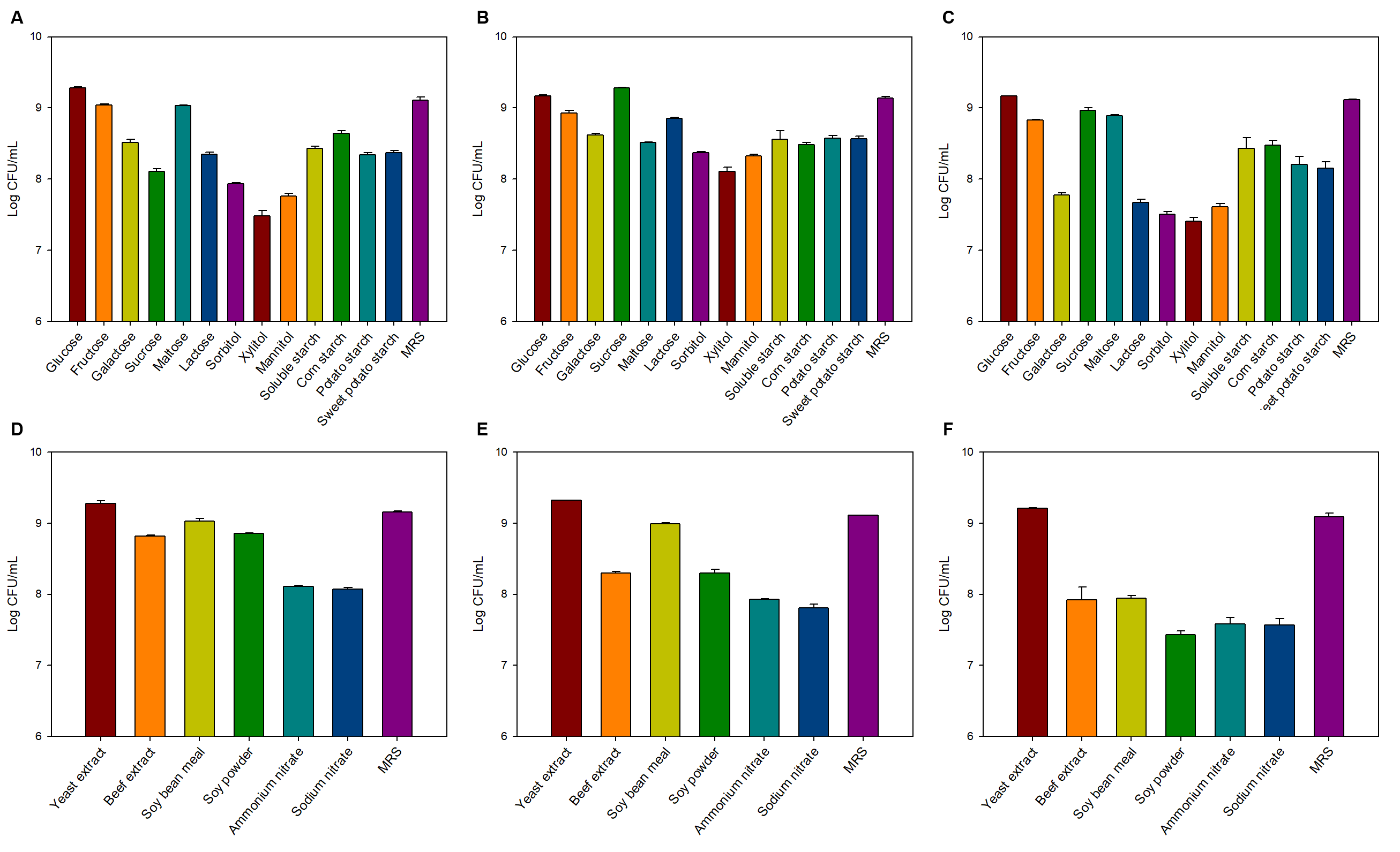

Supplement: S1 Fig — (A) W. cibaria WiKim28. (B) L. sakei WiKim31. (C) L. curvatus WiKim38. (D) W. cibaria WiKim28. (E) L. sakei WiKim31. (F) L. curvatus WiKim38. (TIF) [file pone.0207801.s001.tif]

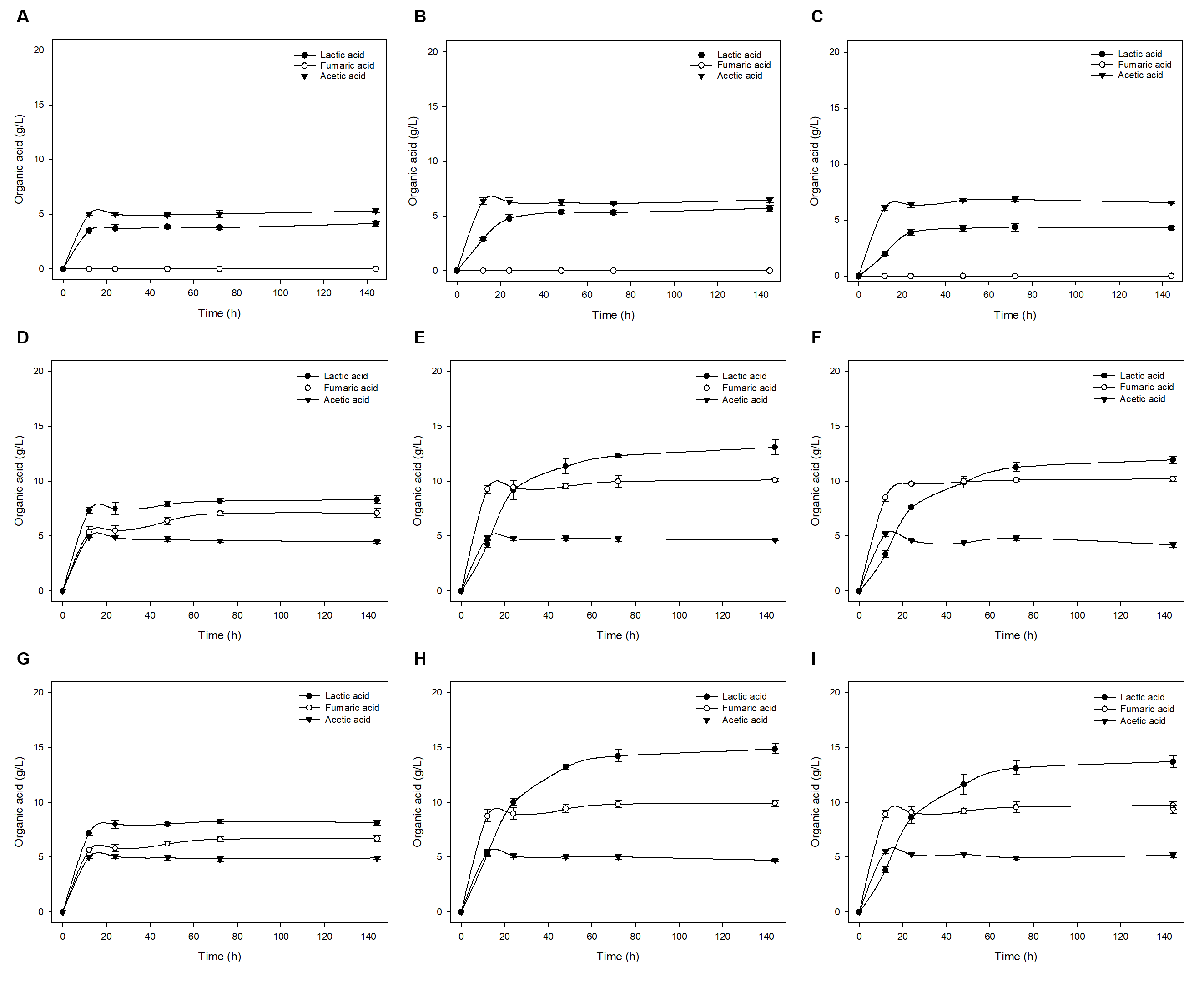

Supplement: S2 Fig — Time courses of organic acid production for the kimchi cabbage waste (KCW) of 1.5, 4.5, and 6.0% (dry matter, w/v). W. cibaria WiKim28 (A) KCW of 1.5%, (B) KCW of 3.0%, (C) KCW of 6.0%. L. sakei WiKim31 (D) KCW of 1.5%, (E) KCW of 3.0%, (F) KCW of 6.0%. L. curvatus WiKim38 (G) KCW of 1.5%, (H) KCW of 3.0%, (I) KCW of 6.0%. (TIF) [file pone.0207801.s002.tif]

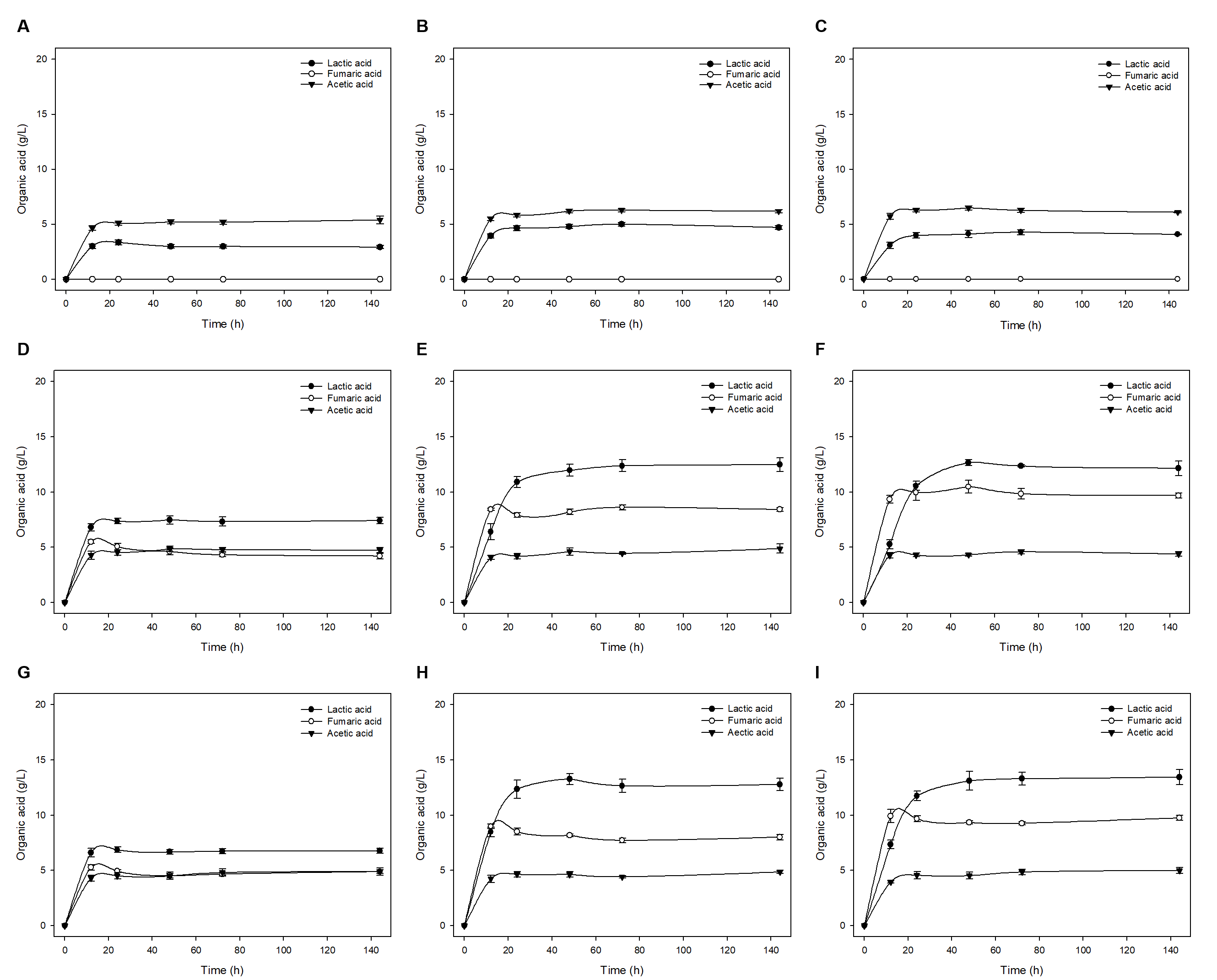

Supplement: S3 Fig — Time courses of organic acid production for the kimchi cabbage waste (KCW) of 1.5, 4.5, and 6.0% (dry matter, w/v). W. cibaria WiKim28 (A) KCW of 1.5%, (B) KCW of 3.0%, (C) KCW of 6.0%. L. sakei WiKim31 (D) KCW of 1.5%, (E) KCW of 3.0%, (F) KCW of 6.0%. L. curvatus WiKim38 (G) KCW of 1.5%, (H) KCW of 3.0%, (I) KCW of 6.0%. (TIF) [file pone.0207801.s003.tif]
