# Supplementary material for: Effective approach to organic acid production from agricultural kimchi cabbage waste and its potential application
Source: PLoS One. 2018 Nov 20;13(11):e0207801. doi: 10.1371/journal.pone.0207801 (PMC6245790; doi:10.1371/journal.pone.0207801)
Supplement: S1 Table — (DOC) [file pone.0207801.s004.doc]

**S1 Table**. Reducing sugar concentration of KCW hydrolysate under different enzyme loading content.

| No. | Pectinase (mg/g KCW) | Cellulase (mg/g KCW) | Reducing sugar (mg/mL) |
| --- | --- | --- | --- |
| 1 | 2.1 | 0 | 4.66 |
| 2 | 2.1 | 2.8 | 5.07 |
| 3 | 2.1 | 5.6 | 5.28 |
| 4 | 2.1 | 11.2 | 5.32 |
| 5 | 2.1 | 22.4 | 5.39 |
| 6 | 4.2 | 0 | 4.81 |
| 7 | 4.2 | 2.8 | 5.16 |
| 8 | 4.2 | 5.6 | 5.45 |
| 9 | 4.2 | 11.2 | 5.50 |
| 10 | 4.2 | 22.4 | 5.62 |
| 11 | 8.4 | 0 | 5.01 |
| 12 | 8.4 | 2.8 | 5.42 |
| 13 | 8.4 | 5.6 | 5.48 |
| 14 | 8.4 | 11.2 | 5.52 |
| 15 | 8.4 | 22.4 | 5.73 |
| 16 | 16.8 | 0 | 5.41 |
| 17 | 16.8 | 2.8 | 5.43 |
| 18 | 16.8 | 5.6 | 5.53 |
| 19 | 16.8 | 11.2 | 5.61 |
| 20 | 16.8 | 22.4 | 5.73 |

Values represent the average of three replicates.
